# Supplementary material for: Impact of Thermally Reducing Temperature on Graphene Oxide Thin Films and Microsupercapacitor Performance
Source: Nanomaterials (Basel). 2022 Jun 28;12(13):2211. doi: 10.3390/nano12132211 (PMC9268352; doi:10.3390/nano12132211)
Supplement: Supplementary file 1 [file nanomaterials-12-02211-s001.zip › nanomaterials-1774322-supplementary.pdf]

nanomaterials

*Supplementary Materials*

# Impact of Thermally Reducing Temperature on Graphene Oxide Thin Films and Microsupercapacitor Performance

**Vusani M. Maphiri, Daba T. Bakhoum, Samba Sarr, Ndeye F. Sylla, Gift Rutavi and Ncholu Manyala ***

Department of Physics, Institute of Applied Materials, SARChI Chair in Carbon Technology and Materials, University of Pretoria, Pretoria 0028, South Africa; vusanimuswamaphiri@gmail.com (V.M.M.); thiogna@yahoo.fr (D.T.B.); ssarr3112@gmail.com (S.S.); ntoufasylla@gmail.com (N.F.S.); rutavigift@yahoo.com (G.R.)

* Correspondence: ncholu.manyala@up.ac.za; Tel.: +27-12-420-3549; Fax: +27-12-420-3546

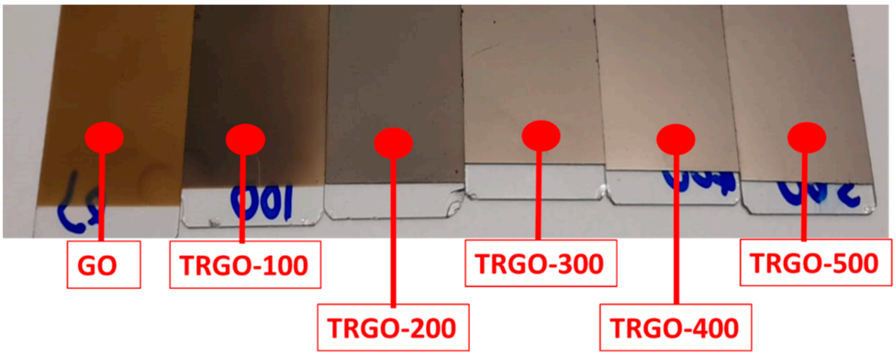


**Figure** S1. The digital images showing the appearance (colour) of the prepared thin film on MSG.

Figure S2a shows the interspacing distance of Graphite, GO and TRGO thin films. The interspacing distance of the TRGO-100 to TRGO-300 could not be calculated because of the diffraction peak of the MSG which has occurred at the same passion as the TRGO. This can be seen on Figure 2 and Figure S1b–d.

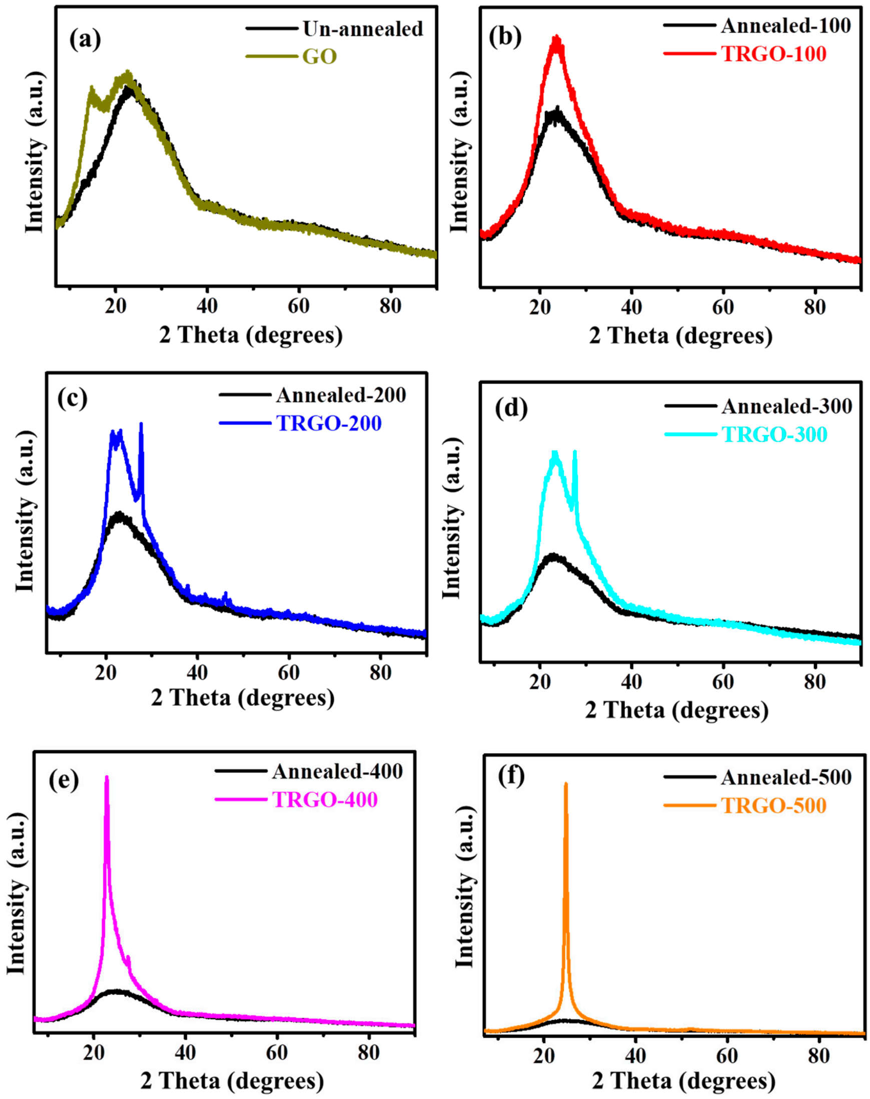


**Figure S2.** The XRD patterns of (a) GO and **(b)–(f)** TRGO-100 to TRGO-500, plotted together with the diffraction pattern of the microscopic glass under the same conditions.

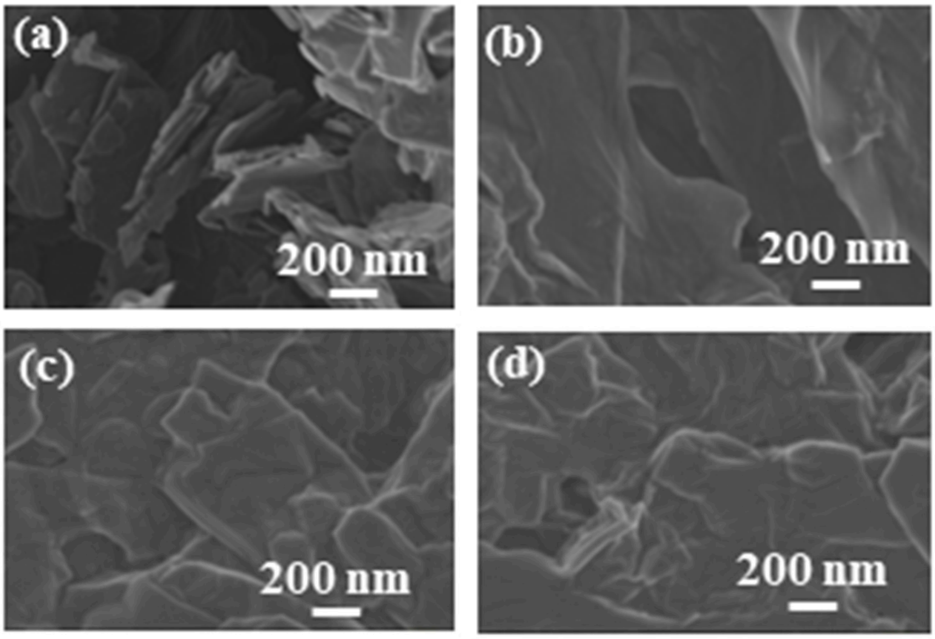


**Figure S3.** SEM images of (a) graphite, (b) GO, (c) TRGO-300 and (d) TRGO-500.

**Surface Topography**

The three-dimensional AFM images of the GO and TRGO samples are displayed in Figure S4 (a)–(f), while the corresponding colour scale is displayed in Fig.ureS4 (g) and the Roughness parameter are displayed on Table S1. The AFM of GO and TRGO-100 shows an average sheet height of approximately 1–1.5 μm due to the presence of the surface oxygen functional group. After the thermal reduction process, it can be easily elucidated that average sheet height has dramatically reduced due to the reduced oxygen content encouraging the van der Waals force through the π–π interaction of the adjacent sheet from restacking. The spike-like feathers at TRGO-200 to TRGO-500 are due to the wrinkle and crumbling of the GO sheets during the TR process.

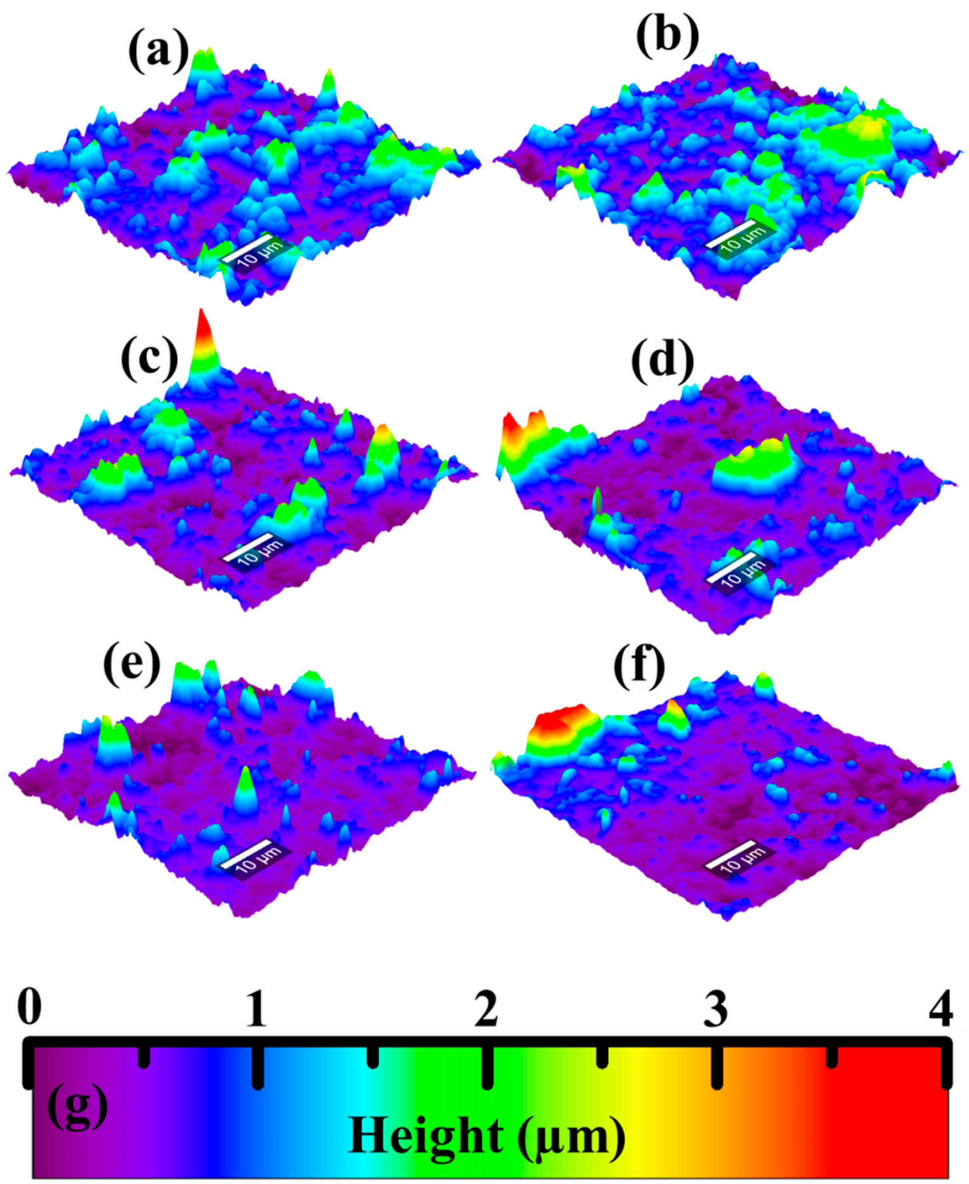


**Figure S4.** The AFM micrograph of **(a)** GO and **(b)–(f)** TRGO-100 to TRGO-500; and **(g)** corresponding height colour scale.

**Table S1.** Roughness parameter of the prepared GO/TRGO thin film.

| Roughness Parameters | GO | TRGO-100 | TRGO-200 | TRGO-300 | TRGO-400 | TRGO-500 |
|---|---|---|---|---|---|---|
| Number of Pixels | 262144 | 262144 | 262144 | 262144 | 262144 | 262144 |
| True Area [µm²] | 3893.59 | 3939.16 | 3886.2 | 3827.2 | 3792.93 | 3803.26 |
| Reference Area [µm²] | 3585.95 | 3585.95 | 3585.95 | 3585.95 | 3585.95 | 3585.95 |
| SDR [%] | 9.11892 | 10.5894 | 9.02563 | 7.27021 | 6.28532 | 6.66457 |
| SDQ [nm] | 0.442367 | 0.47824 | 0.442939 | 0.408333 | 0.369572 | 0.39462 |
| SSC [1/µm] | 0.17374 | 0.191549 | 0.178306 | 0.164417 | 0.158343 | 0.141028 |

**Table S2.** Sample ID, interplanar distance and carbon content.

| Sample ID | Interplanar distance (nm) | Carbon content |
|---|---|---|
| Graphite | 0.3458 | - |
| GO | 0.6019 | 0.068 |
| TRGO-100 | - | 0.97 |
| TRGO-200 | - | 2.26 |
| TRGO-300 | - | 7.05 |
| TRGO-400 | 0.3864 | 9.657 |
| TRGO-500 | 0.3566 | 11.74 |

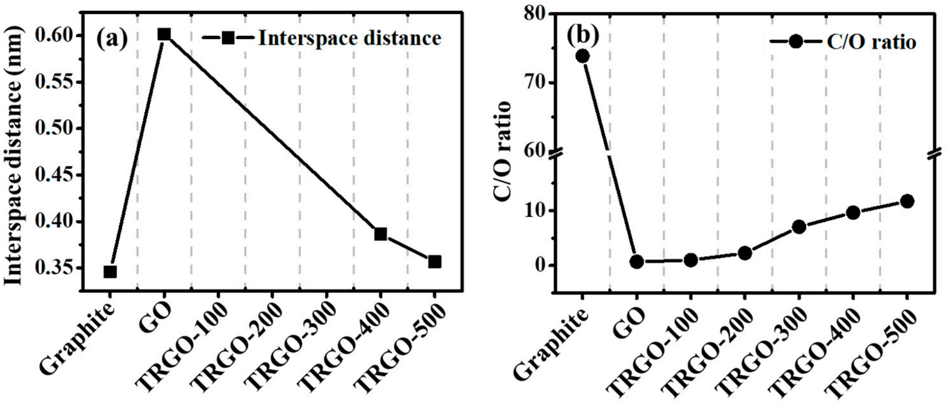


**Figure S5. (a)** Interspace distance and **(b)** C/O ratio as a function for graphite, GO and TRGO-100, TRGO-200, TRGO-300, TRGO-400 and TRGO-500.

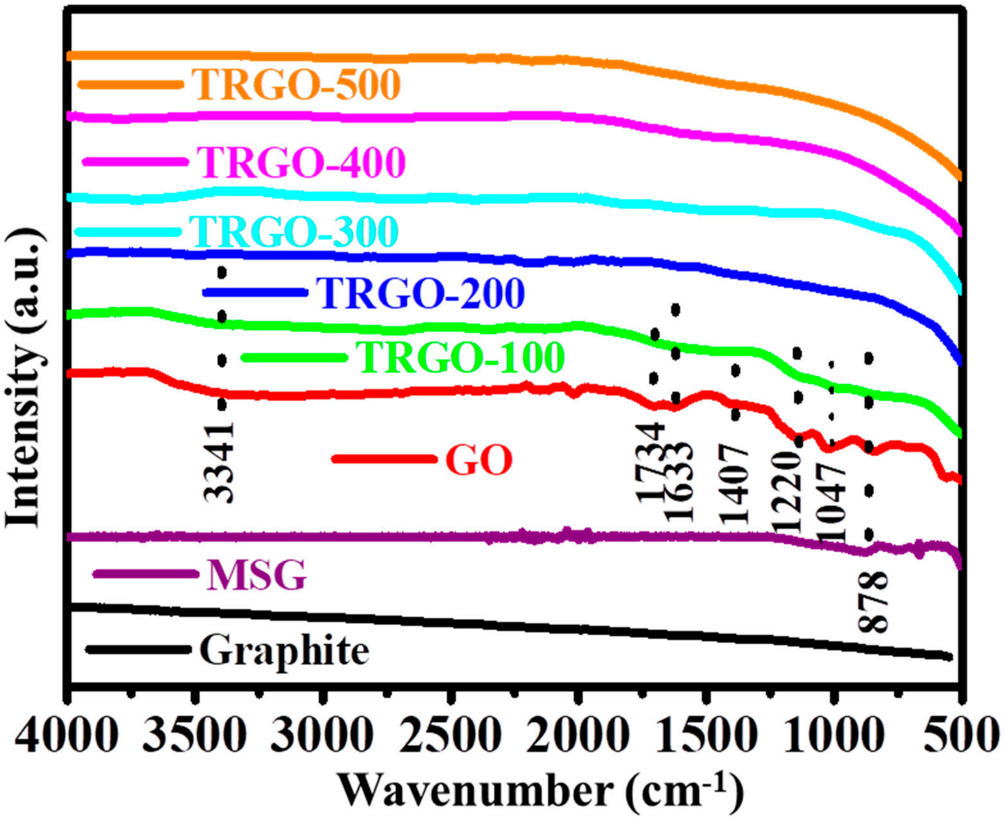

**Figure S6.** The FTIR of graphite, MSG, GO and TRGO samples.

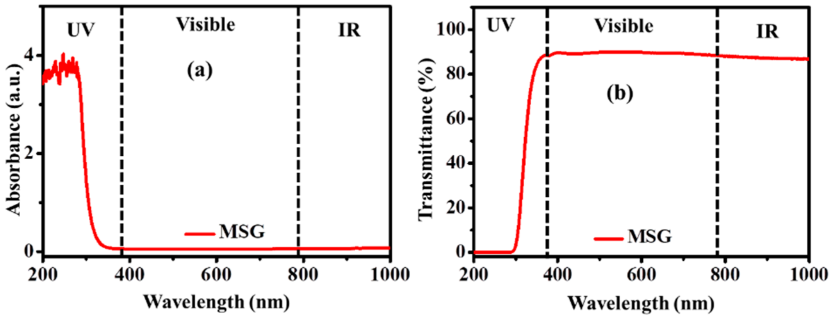


**Figure S7. (a)** Absorbance and **(b)** transmittance of microscope glass.

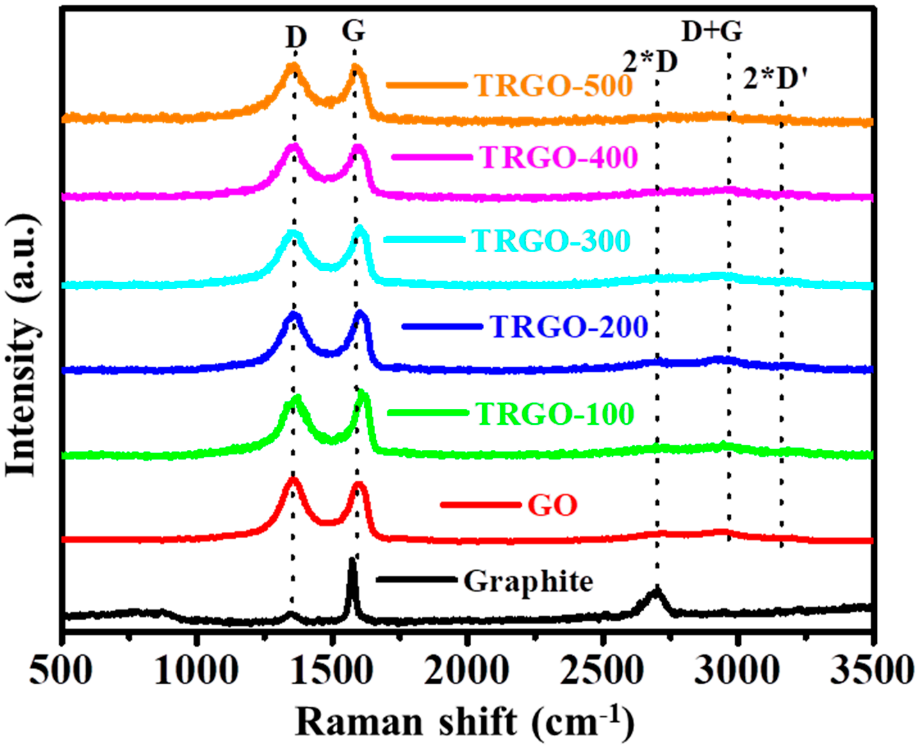


**Figure S8.** Raman spectra of graphite, GO and TRGO-100–TRGO-500.

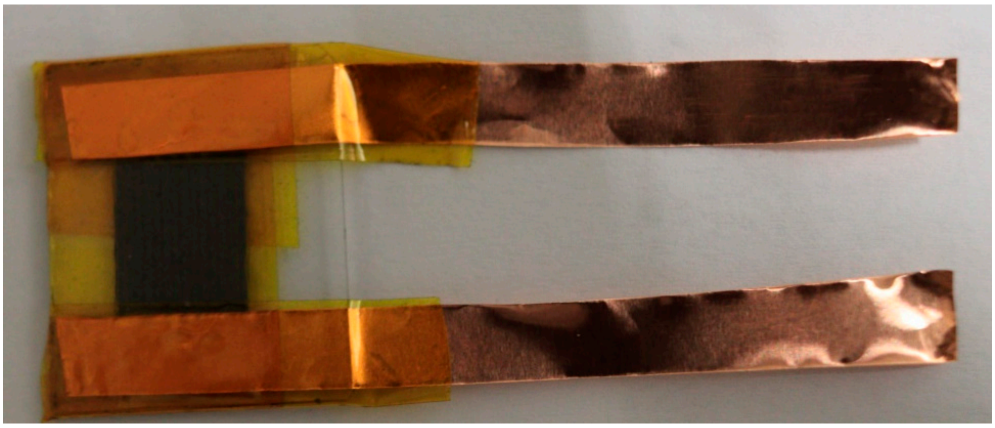

**Figure S9.** Digital image of μ-SC.

**Table S3.** Dimensions and parameters of μ-SC.

| Parameters | μ-SC |
|---|---|
| Number of digits per unit area, $n$ ($cm^{-2}$) | 14 |
| Width, $W$ (mm) | 0.71 |
| Length, $L$ and Breadth, $B$ (mm) | 10 |
| Interspace, $i$(μm) | 38 |
| Edge, $E$ (mm) | 4 |
| Total surface area ($mm^2$) | 100 |

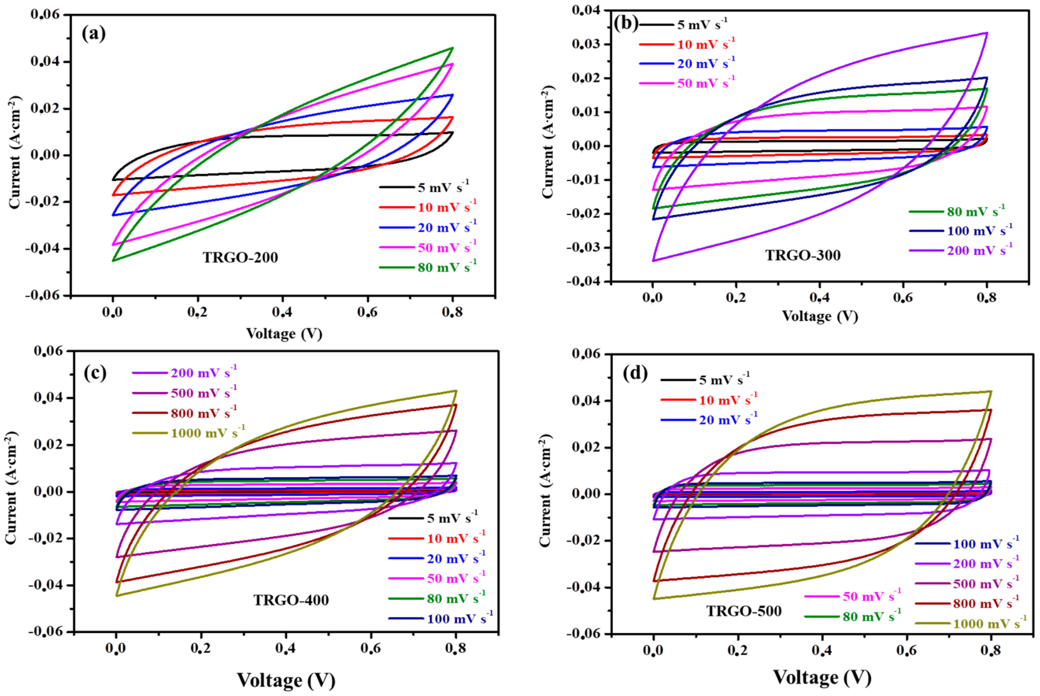


**Figure S10 (a)–(d)** CV curves of TRGO-200–TRGO-500.

The areal capacitance ($C_{\text{Areal}}$) displayed in Figure 8c and Table S4 of the TRGO samples was estimated from the CV curves using Equation (S1) [1–3]

$$C_{\text{Areal}} = \frac{\int_{V_i}^{V_f} i\,dV}{2\upsilon(V_f - V_i)A} \quad \text{(S1)}$$

where $V_f$ and $V_i$ are the final and initial values of the working potential (v), respectively. $i$, υ, and $A$ are the current (mA), various scan rate ($mVs^{-1}$), and total surface area ($cm^2$) of the μ-SC including the interdigit space.

**Table S4.** The areal capacitance determined from the CV curve.

| Scan rate mV $s^{-1}$ | Areal capacitance (mF $cm^{-2}$) | | | |
|---|---|---|---|---|
| | **TRGO-200** | **TRGO-300** | **TRGO-400** | **TRGO-500** |
| 5 | 0.7074 | 0.1451 | 0.0444 | 0.0319 |
| 10 | 0.5421 | 0.1227 | 0.0382 | 0.0298 |
| 20 | 0.3773 | 0.1061 | 0.0348 | 0.0274 |
| 50 | 0.2085 | 0.0865 | 0.0301 | 0.0256 |
| 80 | 0.1498 | 0.0756 | 0.0282 | 0.0244 |
| 100 | - | 0.0695 | 0.0267 | 0.0239 |
| 200 | - | 0.0506 | 0.0234 | 0.0220 |
| 500 | - | - | 0.0179 | 0.0194 |
| 800 | - | - | 0.0148 | 0.0172 |

| | | | | |
|---|---|---|---|---|
| 1000 | - | - | 0.0140 | 0.0159 |
| 1800 | | | | 0.0149 |

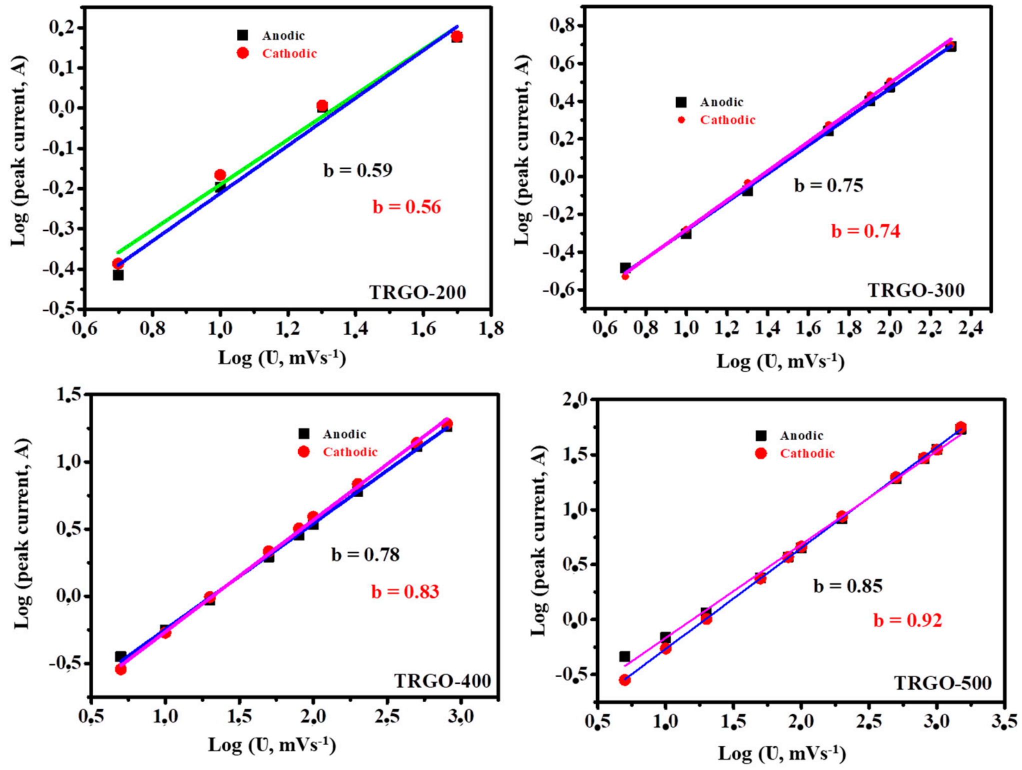


**Figure S11.** log I versus log ʋ curves for the anodic and cathodic currents at **(a)** TRGO-200, **(b)** TRGO-300, **(c)** TRGO-400 and **(d)** TRGO-500 reducing temperatures, respectively.

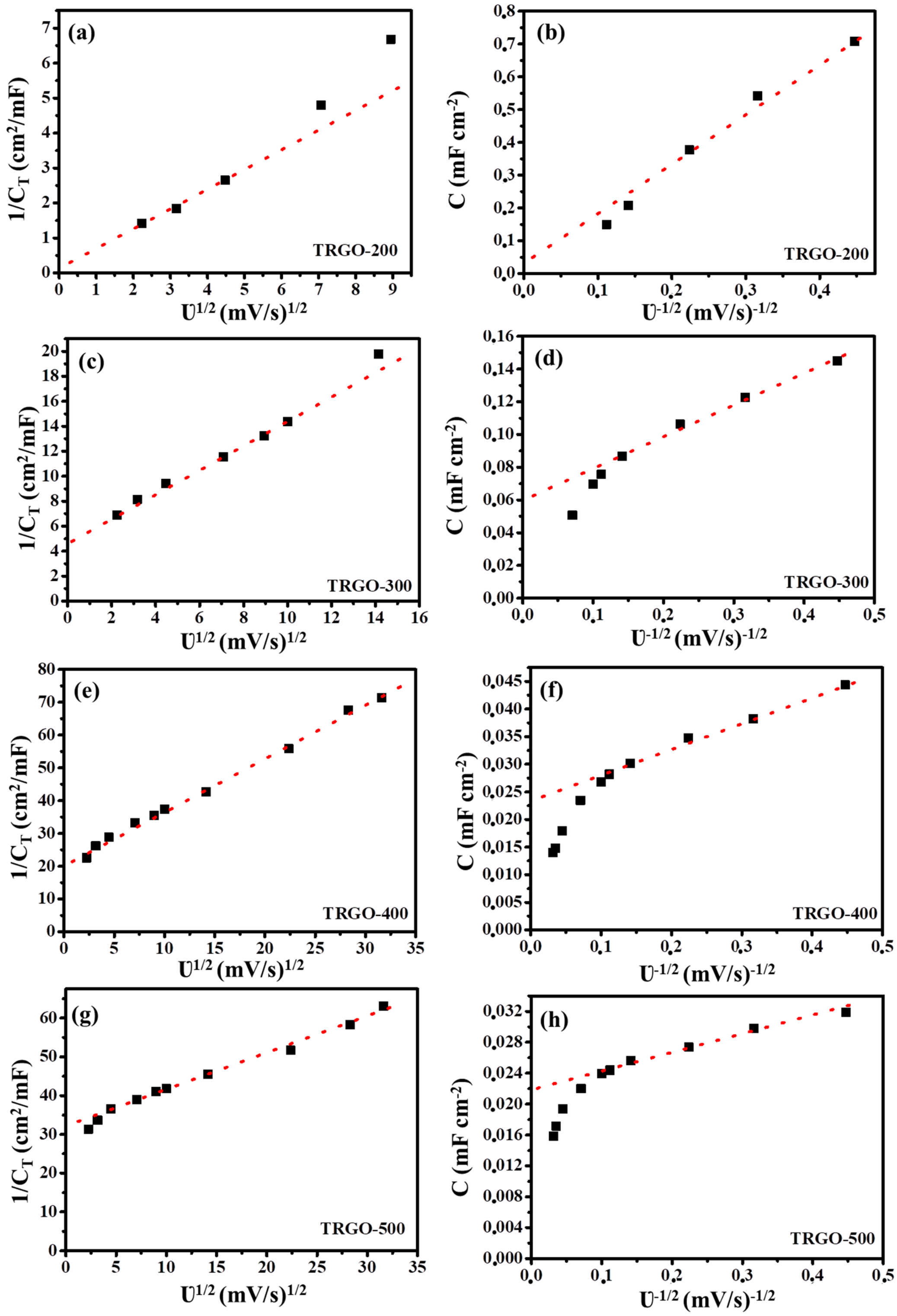


**Figure S12** Trasatti's method for the TRGO micro-supercapacitors: **(a,c,e,g)** inverse capacitance as a function of square root of scan rate and **(b,d,f,h)** capacitance as a function of inverse square root of scan rate.

The Equation (S2) was used to calculate the diffusion ($C_{\text{pseudo}}$) contribution.

$$C_{\text{T}} = C_{\text{EDL}} + C_{\text{pseudo}} \quad \text{(S2)}$$

**Table S5.** Sample ID, maximum total capacitance, maximum EDL capacitance and maximum pseudocapacitance.

| Sample ID | $C_T$ (F g$^{-1}$) | $C_{EDL}$ (F g$^{-1}$) | $C_{pseudo}$ (F g$^{-1}$) |
|---|---|---|---|
| TRGO-200 | 5.6524 | 0.0334 | 5.6190 |
| TRGO-300 | 0.2058 | 0.0613 | 0.1445 |
| TRGO-400 | 0.0497 | 0.0236 | 0.0261 |
| TRGO-500 | 0.0311 | 0.0222 | 0.0089 |

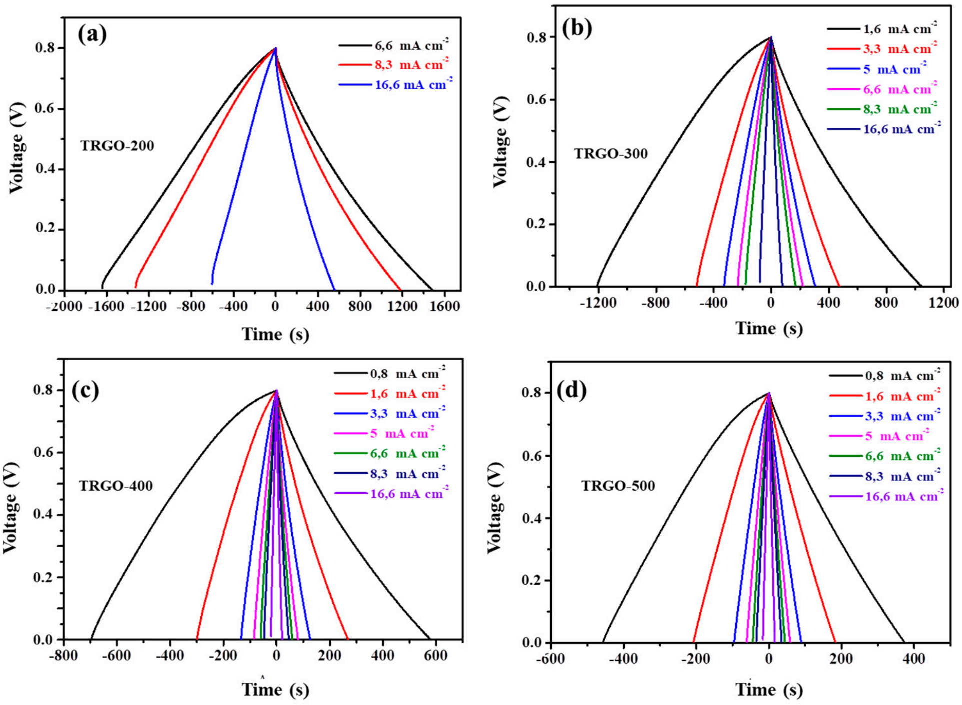


**Figure S13. (a)–(d)** GCD curves of TRGO-200–TRGO-500.

**Table S6.** The areal capacitance determined from the GCD curve.

| Current density | Areal capacitance (mF cm$^{-2}$) | | | |
|---|---|---|---|---|
| (μA cm$^{-2}$) | TRGO-200 | TRGO-300 | TRGO-400 | TRGO-500 |
| 0.083 | - | - | 0.0600 | 0.0387 |
| 0.16 | - | 0.2143 | 0.0536 | 0.0366 |
| 0.33 | - | 0.1952 | 0.0517 | 0.0367 |
| 0.5 | - | 0.1904 | 0.0501 | 0.0364 |
| 0.66 | 1.2255 | 0.1809 | 0.0481 | 0.0356 |
| 0.83 | 1.2262 | 0.1754 | 0.0472 | 0.0354 |
| 1.66 | 1.1569 | 0.1506 | 0.0434 | 0.0338 |

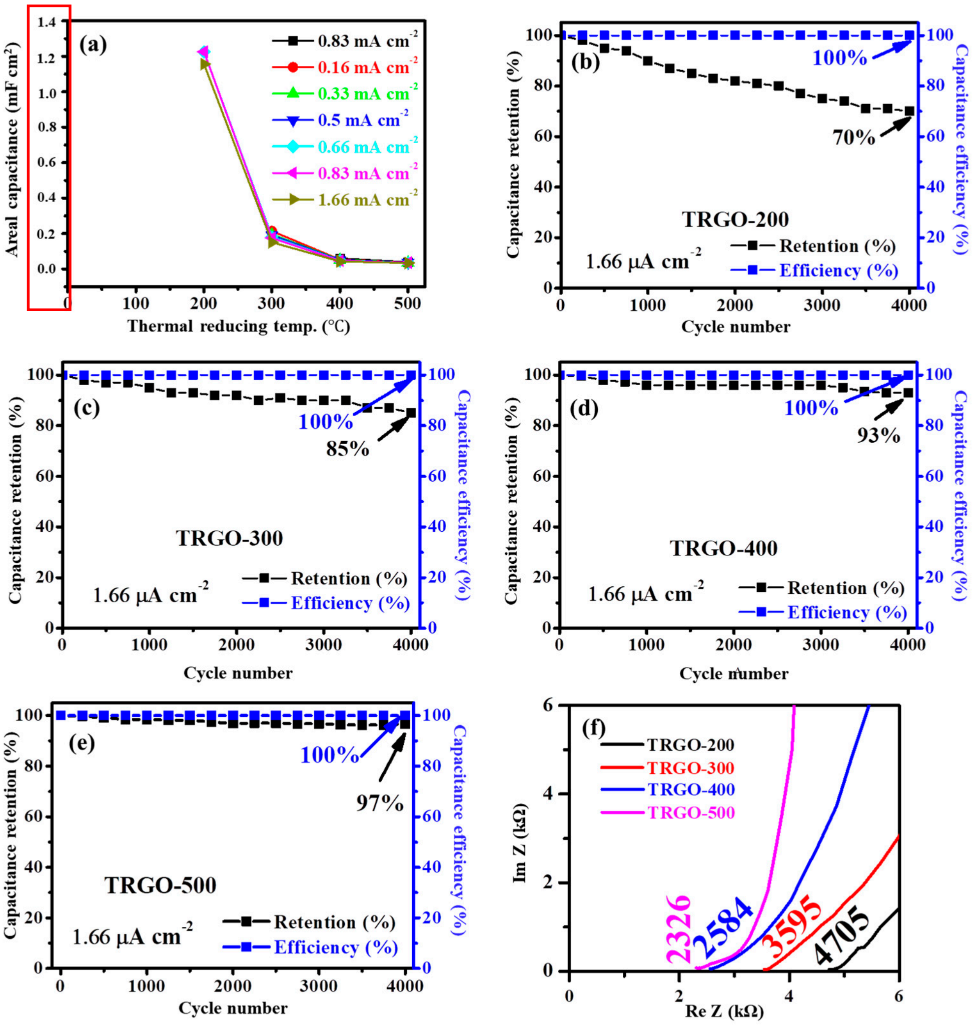


**Figure S14. (a)** Area capacitance as a function of reducing temperature at various current densities, **(b)–(e)** the capacitance retention and capacitance columbic efficiency of the TRGO sample and **(f)** EIS Nyquist plot at high frequency region.

The areal energy ($E_{\text{areal}}$) and power ($P_{\text{areal}}$) calculated from the below Equations (S3) and (S4)

$$E_{\text{areal}} = \frac{1}{3600\Gamma}\int iVdt \quad \text{(S3)}$$

$$P_{\text{areal}} = 3600 \times \frac{E_{areal}}{\Delta t} \quad \text{(S4)}$$

where Γ, $\int iVdt$ and $\Delta t$ are the area or volume of the µ-SC, the integral of the discharge curve and discharge time, respectively.

**Table S7.** Sample ID, resistance electrolyte plus charge transfer resistance ($R_e + R_{ct}$), Warburg co-efficient ($\sigma_w$) and ion diffusion coefficient (D).

| Sample ID | Maximum specific power (W cm$^{-2}$) | Maximum specific energy (W h cm$^{-2}$) | $R_e + R_{ct}$ (Ω) | $\sigma_w$ (Ω cm$^2$ s$^{-0.5}$) | D (cm$^2$ s$^{-1}$) |
| --- | --- | --- | --- | --- | --- |

| | | | | | |
|---|---|---|---|---|---|
| TRGO-200 | $3.4006\times10^{-5}$ | 0.5024 | 4.71236 | 12.4831±0.002 | $4.3075\times10^{-13}$ |
| TRGO-300 | $4.4506\times10^{-5}$ | 0.0183 | 3.52368 | 14.6329±0.001 | $3.1351\times10^{-13}$ |
| TRGO-400 | $6.1950\times10^{-5}$ | 0.0044 | 2.53551 | 21.8812±0.001 | $1.4021\times10^{-13}$ |
| TRGO-500 | $6.8788\times10^{-5}$ | 0.0028 | 2.26279 | 37.7070±0.002 | $4.7214\times10^{-14}$ |

The impedance ($Z(\omega)$) is expressed by Equation (S5)

$$Z(\omega) = \frac{1}{j\omega \times C(\omega)} \tag{S5}$$

Therefore,

$$Re\ C = \frac{Im\ Z(\omega)}{\omega|Z(\omega)|^2} \tag{S6}$$

$$Im\ C = \frac{Re\ Z(\omega)}{\omega|Z(\omega)|^2} \tag{S7}$$

where $Re\ Z(\omega)$ and $Im\ Z(\omega)$ are defined as

$$|Z(\omega)|^2 = Re\ Z(\omega)^2 + Im\ Z(\omega)^2 \tag{S8}$$

And

$$\omega = 2\pi f \tag{S9}$$

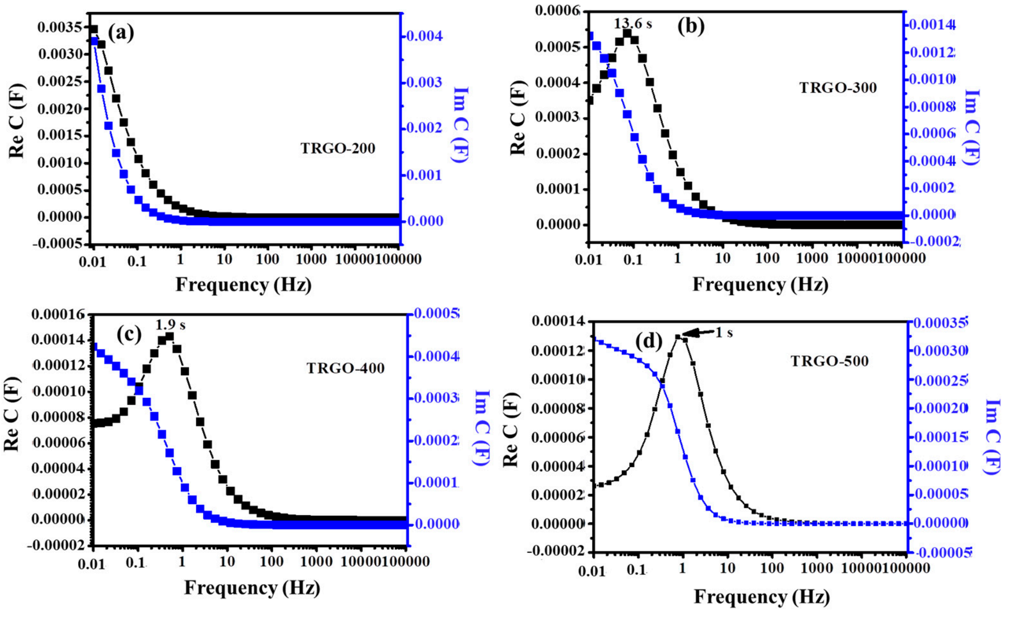


**Figure S15.** The frequency dependence of the Re C and Im C for **(a)–(b)** TRGO-200 to TRGO-500.

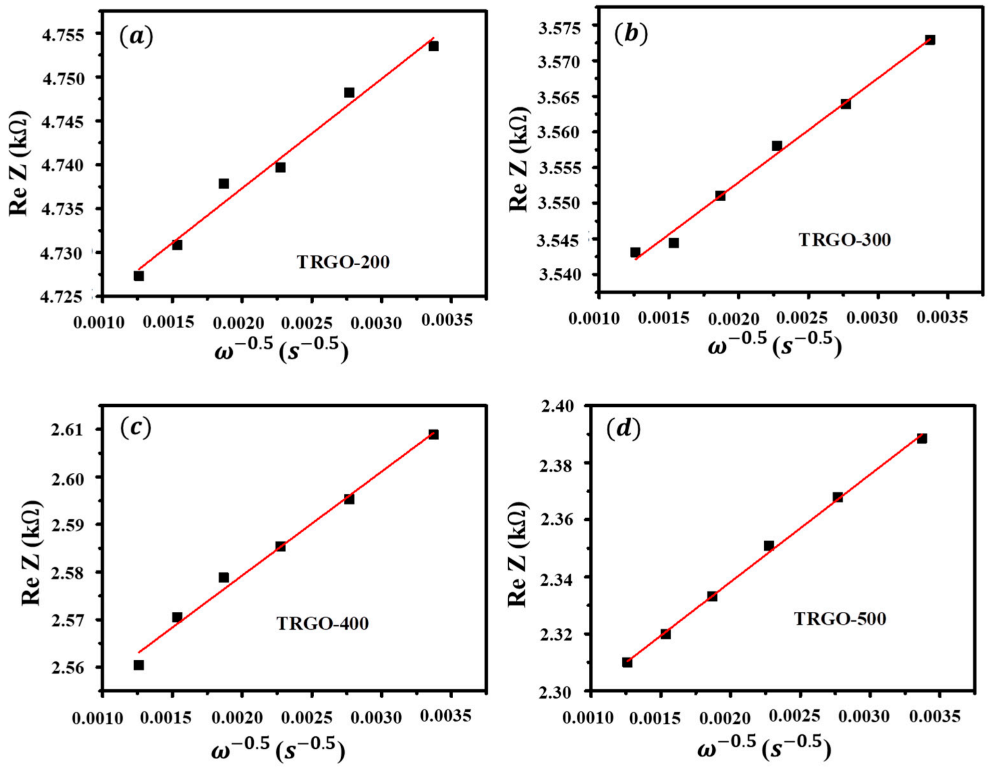


**Figure S16.** The relationship between Re Z and $\omega^{-0.5}$ at low frequencies for TRGO samples

Equation (S10) [4] was used to calculate ion diffusion coefficient (D)

$$D = 0.5\left(\frac{RT}{AF^2\sigma_w C}\right)^2 \quad \text{(S10)}$$

where $R$ is the gas constant (8.314 J mol$^{-1}$ K$^{-1}$), $T$ is the temperature (298.5 K), A is the total area (cm$^2$) of the μ-SC fingers, $F$ is the Faraday's constant (96,500C mol$^{-1}$) and $C$ is the molar concentration of $H^+$ ions calculated using Equations (S11) and (S12).

The proton concentration $[H^+]$ of the used PVA/$H_3PO_4$ electrolyte was calculated using Equations (S11) and (S12).

$$Ph = -\log[H^+] \quad \text{(S11)}$$

$$[H^+] = 10^{-Ph} \quad \text{(S12)}$$

where, the $Ph$ was measured to be 1.64 corresponding to $[H^+]$ of 0.023

## Reference


1. Maphiri, V.M.; Rutavi, G.; Sylla, N.F.; Adewinbi, S.A.; Fasakin, O.; Man, N. Novel Thermally Reduced Graphene Oxide Microsupercapacitor Fabricated via Mask—Free AxiDraw Direct Writing. *Nanomaterials* **2021**, *11*, 1909.
2. Liu, L.; Ye, D.; Yu, Y.; Liu, L.; Wu, Y. Carbon-based flexible micro-supercapacitor fabrication via mask-free ambient micro-plasma-jet etching. *Carbon N. Y.* **2017**, *111*, 121–127. https://doi.org/10.1016/j.carbon.2016.09.037.
3. Li, J.; Levitt, A.; Kurra, N.; Juan, K.; Noriega, N.; Xiao, X.; Wang, X.; Wang, H.; Alshareef, H.N.; Gogotsi, Y. MXene-conducting polymer electrochromic microsupercapacitors. *Energy Storage Mater.* **2019**, *20*, 455–461. https://doi.org/10.1016/j.ensm.2019.04.028.
4. Cui, Y.; Zhao, X.; Guo, R. Improved electrochemical performance of La0.7Sr0.3MnO3 and carbon co-coated LiFePO4 synthesized by freeze-drying process. *Electrochim. Acta* **2010**, *55*, 922–926. https://doi.org/10.1016/J.ELECTACTA.2009.08.020.
